# High visceral fat percentage is associated with poor outcome in endometrial cancer

**DOI:** 10.18632/oncotarget.21917

**Published:** 2017-10-19

**Authors:** Karen Klepsland Mauland, Øyvin Eng, Sigmund Ytre-Hauge, Ingvild L. Tangen, Anna Berg, Helga B. Salvesen, Øyvind O. Salvesen, Camilla Krakstad, Jone Trovik, Erling A. Hoivik, Henrica Maria Johanna Werner, Gunnar Mellgren, Ingfrid S. Haldorsen

**Affiliations:** ^1^ Centre for Cancer Biomarkers, CCBIO, Department of Clinical Science (K2), University of Bergen, Bergen, Norway; ^2^ Department of Gynecology and Obstetrics, Haukeland University Hospital, Bergen, Norway; ^3^ Hormone Laboratory, Haukeland University Hospital, Bergen, Norway; ^4^ Department of Radiology, Haukeland University Hospital, Bergen, Norway; ^5^ Department of Clinical Medicine (K1), University of Bergen, Bergen, Norway; ^6^ Unit for Applied Clinical Research, Department of Public Health and Nursing, Norwegian University of Science and Technology, Trondheim, Norway; ^7^ Centre for Cancer Biomarkers, CCBIO, Department of Biomedicine, University of Bergen, Bergen, Norway; ^8^ KG Jebsen Centre for Diabetes Research, Department of Clinical Science (K2), University of Bergen, Bergen, Norway

**Keywords:** endometrial cancer, obesity, visceral fat, hormone receptor expression, transcriptional profiling

## Abstract

Despite evidence of increased endometrial cancer (EC) risk in obese women, the impact of obesity on clinical and histological phenotype is poorly understood. This study explored abdominal fat volumes and fat distribution quantified by computed tomography (CT), in relation to tumor characteristics and outcome. 227 EC patients with preoperative abdominal CT scans were included. Total abdominal fat volume (TAV), subcutaneous abdominal fat volume (SAV) and visceral abdominal fat volume (VAV) were quantified, and visceral fat percentage calculated (VAV%=[VAV/TAV]x100). Waist circumference (WC) and liver density (LD) were measured, and body mass index (BMI) calculated. Data for estrogen, progesterone and androgen receptor (ERα/PR/AR) expression by immunohistochemistry were available for 149 tumors, and global gene expression data for 105 tumors. High BMI, TAV, SAV, VAV and WC, and low LD, were associated with low grade endometrioid tumors and PR and AR positivity (all p≤0.03). High VAV% was associated with high age (p<0.001), aneuploidy (p=0.01) and independently predicted reduced disease-specific survival (HR 1.05, 95% CI 1.00-1.11, p=0.041). Tumors from patients with low VAV% showed enrichment of gene sets related to immune activation and inflammation. In conclusion, high VAV% independently predicts reduced EC survival. Tumors arising in patients with low VAV% show enrichment of immune and inflammation related gene sets, suggesting that the global metabolic setting may be important for tumor immune response.

## INTRODUCTION

Excess body weight, typically measured as high body mass index (BMI; kg/m^2^), is a major risk factor for endometrial cancer (EC) development [1, 2]. Despite the well-known association between obesity and increased risk of various cancers, the underlying mechanisms linking obesity to cancer development are complex, and only partly understood [3, 4]. Unopposed endogenous estrogen signaling is known to promote EC development [5], which could explain some of the risk attributed to obesity since estrogens are primarily produced in the adipose tissue after menopause [3]. However, obesity is an independent risk factor for both type 1 and type 2 EC [6], although the latter is generally considered less hormone dependent [7]. Other putative mechanisms involved in obesity-related carcinogenesis include increased inflammatory signaling through various mediators, and increased levels of insulin and insulin like growth factor 1 (IGF1) [3–5, 8], thought to promote proliferation, production of anti-apoptotic signals, local inflammation and angiogenesis.

BMI as a surrogate marker for obesity has met considerable criticism. Although easily calculated and monitored in the clinic, it is a crude parameter not distinguishing between fat and muscle mass [9]. Furthermore, neither BMI nor other anthropometric measures including waist circumference or hip/waist ratio account for the localization of abdominal fat deposits in the subcutaneous or visceral compartments. Individuals with high quantities of visceral fat, due to increased mesenteric, omental and retroperitoneal fat storage, carry an increased risk of cardiovascular disease and type 2 diabetes [10]. They are also at higher risk of developing breast-, colorectal- and esophageal cancer [11], compared to individuals with less visceral fat. The visceral fat exerts distinct endocrine activity, which is thought to contribute to its increased pathogenic potential [10]. There is also emerging evidence that the fat distribution pattern may be associated with survival and therapeutic response in several cancer types, i.e. malignant melanomas, breast-, colorectal- and esophageal cancers [12–15]. Assessment of the abdominal fat compartments by abdominal computed tomography (CT) or magnetic resonance imaging (MRI) has been shown to be both feasible and reliable [16–18]. Furthermore, abdominal CT and MRI may allow estimation of hepatic steatosis [19], considered to be closely linked to obesity [3, 20].

Only a few studies have previously explored the visceral and subcutaneous fat distribution in relation to clinicopathological characteristics in EC [21, 22]. One study suggested an association between a high proportion of visceral fat and aggressive clinical features, including lymph node metastasis and extrauterine disease [22]. However, none of these studies reported volumetric estimates of the abdominal fat compartments, nor included patient survival data.

This study aimed to explore CT-quantified abdominal fat volumes and fat distribution, as well as CT-assessed abdominal circumference and hepatic attenuation in relation to BMI, clinicopathological features and survival in a large endometrial cancer patient series. Furthermore, we aimed to explore the CT-derived measures in relation to molecular markers in corresponding tumor tissue.

## RESULTS

### Intraabdominal fat volumes are correlated with BMI

**Figure 1:**
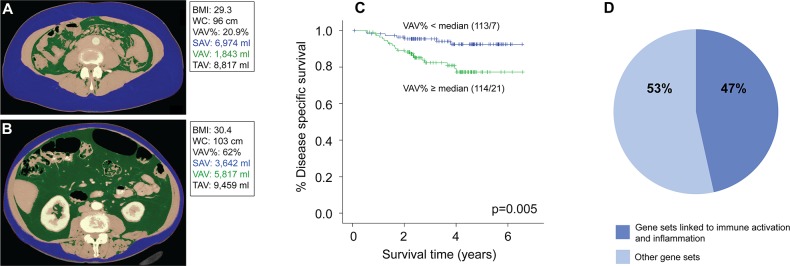
Low visceral fat percentage is associated with better disease-specific survival and enrichment of immune and inflammation related gene sets in endometrial tumors Abdominal CT scans with segmentation of subcutaneous and visceral fat compartments in two patients with comparable BMI measurements, but with different visceral fat percentage (VAV%): **(A)** Patient with BMI 29 and low visceral fat percentage (VAV%=21%). **(B)** Patient with BMI 30 and high visceral fat percentage (VAV%=62%). **(C)** Kaplan-Meier curve showing significantly reduced disease-specific survival in patients with high VAV% (median cut-off: ≥37%; p=0.005, log-rank test). **(D)** Gene set enrichment analysis of tumors with low (<37%, n=56) versus high (≥37%, n=49) VAV% was performed. Diagram shows the percentage of Hallmark and Gene ontology (GO) gene sets enriched in tumors with low VAV% that were linked to immunogenic and inflammatory pathways versus other pathways. Cut-off for selected gene sets was False Discovery Rate (FDR) <5%. A full description of the gene sets is supplied in Supplementary Table 2.

The clinicopathological characteristics of the patient series (n=227) are summarized in Table 1. The mean age was 67 years (range 30-89). The correlations between BMI and the CT-assessed obesity variables (subcutaneous abdominal fat volume, SAV; visceral abdominal fat volume, VAV; total abdominal fat volume, TAV; [VAV/TAV]x100, VAV%; waist circumference, WC; and liver density, LD) are given in Table 2. All the CT estimated fat volumes (TAV, VAV and SAV), as well as WC (Figure 1A and 1B), were strongly positively correlated with patient BMI and with each other (Table 2), whereas LD was strongly negatively correlated with all obesity markers (Table 2, Supplementary Figure 1). The visceral fat percentage (VAV%) was not correlated with BMI, WC or TAV, but was weakly negatively correlated with LD (Table 2).

**Table 1:** Clinicopathological characteristics of 227 included endometrial cancer patients

	n (%)
**Primary treatment**	
HBSO	221 (97)
Curettage/palliative surgery	6 (3)
**FIGO stage**	
Stage I	180 (79)
Stage II	21 (9)
Stage III	21 (9)
Stage IV	5 (2)
**Histological subtype & grade** *(n=225)*	
Endometrioid grade 1-2	152 (68)
Endometrioid grade 3	32 (14)
Non-endometrioid	41 (18)
**Menopausal status**	
Pre/perimenopausal	20 (9)
Postmenopausal	207 (91)
**Type II diabetes**	
No	206 (91)
Yes	21 (9)
**Parity**	
Nulliparous	28 (12)
≥ 1	199 (88)
**Lymphadenectomy**	
Pelvic	154 (68)
Pelvic + para-aortic	24 (11)
No	49 (22)
**Lymph node metastasis** *(n=178)*	
No	159 (99)
Yes	19 (11)
**Ploidy status** *(n=119)*	
Diploid	94 (79)
Aneuploid	25 (21)
**BMI** *(n=226)*	
Mean (SD)	27.9 (5.8)
**Age**	
Mean (SD)	66.9 (11.2)

Abbreviations: BMI: Body mass index; FIGO: International federation of gynecology and obstetrics; HBSO: Hysterectomy with bilateral salpingo-oophorectomy; n: number of patients in each category; SD: Standard deviation.

**Table 2:** Correlations (Spearman rho, ρ) between BMI and CT estimates of obesity in 227 endometrial cancer patients

	Mean ± SD (range), unit	BMI	WC	TAV	VAV	SAV	VAV%
**BMI^1^**	27.9 ± 5.8 (15.6 − 50.0), kg/m^2^						
**Waist circumference (WC, L3-L4 level)**	95.7 ± 13.9 (63 - 135), cm	0.90^**^					
**Total abdominal fat volume (TAV)**	9,534 ± 4,599 (782 - 26,420), ml	0.89^**^	0.91^**^				
**Visceral abdominal fat volume (VAV)**	3,549 ± 1,842 (491 - 9,825), ml	0.78^**^	0.81^**^	0.91^**^			
**Subcutaneous abdominal fat volume (SAV)**	5,984 ± 3,057 (291 - 18,309), ml	0.87^**^	0.89^**^	0.97^**^	0.78^**^		
**Visceral fat percentage (VAV%)**	37.2 ± 8.3 (18.1 - 63.3), %	−0.16	−0.04	0.05	0.42^**^	−0.17^*^	
**Liver density (LD)^2^**	98.7 ± 20.5 (28 - 144), HU	−0.71^**^	−0.70^**^	−0.73^**^	−0.71^**^	−0.67^**^	−0.16^*^

Abbreviations: BMI: Body mass index; CT: Computed tomography; HU: Hounsfield units; SD: Standard deviation.

^*^: Correlation significant at p<0.05 level; ^**^: Correlation significant at p<0.001 level.

^1^ BMI data missing for one patient; ^2^ Liver density data missing for two patients.

### Obesity is associated with low-grade endometrial cancer and PR and AR expression, but not ERα expression

**Figure 2:**
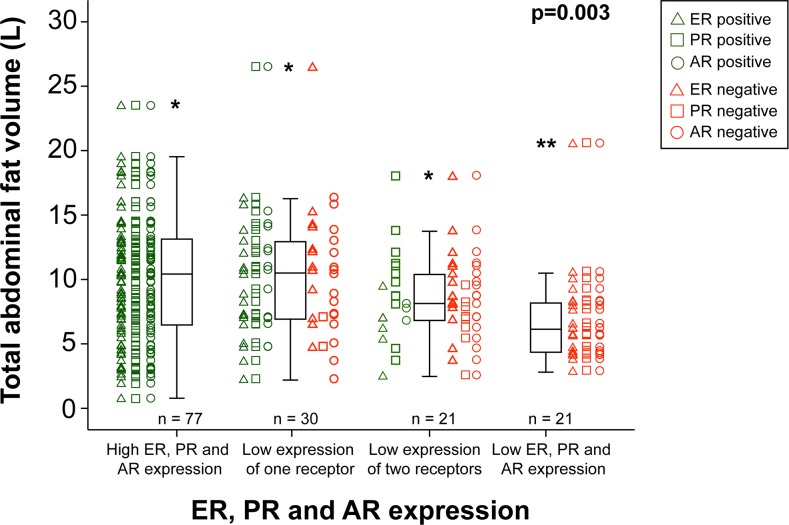
Total abdominal fat volume in relation to hormone receptor status Whisker plots displaying the distribution of total abdominal fat volumes (L) for 149 endometrial cancer patients. From left to right: tumors with high immunohistochemical expression of the estrogen receptor (ER), progesterone receptor (PR) and androgen receptor (AR) (n=77), low expression of one of the receptors (n=30), low expression of two of the receptors (n=21) and low expression of all three receptors (n=21). For each case, positive/negative status for ER, PR or AR is indicated by green/red triangles, squares and circles, respectively. ^*^ outlier > 1.5 interquartile range, ^**^ outlier > 3 interquartile range. P-value: Jonckheere-Terpstra trend test.

High values for the CT estimates reflecting obesity, i.e. TAV, VAV, SAV and WC, and high BMI, were all significantly associated with low-grade endometrioid subtype, and positivity for progesterone (PR) and androgen (AR) receptor in tumor tissue (Table 3). No association was observed between estrogen receptor alpha (ERα) expression and the same obesity measures (Table 3). Exploring the association between loss of hormone receptors with total abdominal fat volume, an incremental decrease in fat volume was noted for patients with loss of one, two and all three receptors (p=0.003; Figure 2). A similar pattern was seen when analyzing endometrioid tumors only (p=0.02), however the tendency was stronger in the subgroup of grade 3 tumors (n=21, p=0.041) compared to grade 1-2 tumors (n=94, p=0.19, data not shown). BMI and subcutaneous fat volume were also significantly higher in younger patients (p=0.04 and p=0.02, respectively; Table 3). In contrast, VAV% was significantly higher in patients with high age (p<0.001) and aneuploid tumors (p=0.01). Subset analysis in the endometrioid subgroup (n=186), revealed a similar overall pattern with low grade disease and PR/AR, but not ERα, positivity significantly associated with higher BMI, WC, VAV, SAV, TAV and LD (Supplementary Table 1).

**Table 3:** BMI and CT-estimated obesity parameters in relation to clinicopathological factors and hormone receptor status for 227 endometrial cancer patients

		BMI		WC		TAV		VAV		SAV		VAV%		LD	
	n (%)	median	p	median	p	median	p	median	p	median	p	median	p	median	p
		(kg/m^2^)		(cm)		(ml)		(ml)		(ml)		(%)		(HU)	
**Histological subtype & grade** *(n=225)*			**0.02**		**0.02**		**0.01**		**0.03**		**0.01**		0.54		**0.01**
Endometrioid grade 1-2	152 (68)	28.0		98		10,302		3,647		6,032		37		99	
Endometrioid grade 3	32 (14)	25.2		87		6,645		2,241		4,310		36		109	
Non-endometrioid	41 (18)	25.6		91		7,978		2,849		4,476		39		106	
**FIGO stage**			0.63		0.74		0.90		0.79		0.66		0.21		0.57
I+II	201 (89)	26.1		96		8,984		3,390		5,613		37		102	
III+IV	26 (11)	26.7		96		8,596		3,160		4,890		40		102	
**Ploidy** *(n=119)*			0.29		0.30		0.37		0.98		0.19		**0.01**		0.46
Diploid	94 (79)	26.4		96		9,438		3,439		5,716		37		99	
Aneuploid	25 (21)	26.1		92		8,024		3,295		4,476		42		102	
**Age (median)**			**0.04**		0.15		0.35		0.21		**0.02**		**<0.001**		0.17
< 67 years	108 (47)	26.8		96		8,920		3,159		6,084		34		101	
≥ 67 years	121 (53)	26.4		95		9,174		3,748		5,041		40		102	
**ERα expression, IHC** *(n=170)*			0.36		0.15		0.20		0.23		0.27		0.99		0.10
Positive	114 (67)	27.1		98		9,627		3,464		5,693		37		100	
Negative	56 (33)	26.0		94		8,152		2,951		4,820		39		105	
**PR expression, IHC** *(n=170)*			**<0.001**		**<0.001**		**<0.001**		**0.001**		**<0.001**		0.37		**0.002**
Positive	131 (77)	28.0		98		10,386		3,596		6,155		37		100	
Negative	39 (23)	24.6		88		6,180		2,273		3,642		39		106	
**AR expression, IHC** *(n=169)*			**0.01**		**0.002**		**0.004**		**0.004**		**0.01**		0.47		**0.01**
Positive	107 (63)	28.0		98		10,420		3,831		6,120		37		98	
Negative	62 (37)	25.1		91		7,957		2,811		4,557		38		106	

Abbreviations: AR: Androgen receptor; BMI: Body mass index; CT: Computed tomography; ERα: Estrogen receptor alpha; FIGO: International federation of gynecology and obstetrics; HU: Hounsfield units; IHC: Immunohistochemistry; LD: Liver density; PR: Progesterone receptor; p: p-values (Independent samples Kruskal-Wallis test for histological subtype and grade, remaining p-values: Mann-Whitney U test); SAV: Subcutaneous abdominal fat volume; TAV: Total abdominal fat volume; VAV: Visceral abdominal fat volume; VAV%: Visceral fat percentage; WC: Waist circumference.

BMI: data missing for one patient.

Liver density: data missing for two patients.

### A high percentage of visceral fat is associated with reduced endometrial cancer survival

None of the volumetric CT variables (TAV, VAV or SAV), BMI, WC or LD (Figure 1A and 1B, Supplementary Figure 1) were significantly associated with survival in univariable Cox analysis (data not shown). However, increasing VAV% was significantly associated with reduced survival in the univariable Cox-model (HR 1.07, 95% CI 1.03 – 1.12, p=0.001; Table 4), also visualized in the Kaplan-Meier plot where patients with VAV% above median (≥37%) had significantly reduced survival (p=0.005, Figure 1C). The same trend was found in analyses stratified for BMI above and below 30, respectively (p=0.04 for both groups; Supplementary Figure 2A and 2B). When adjusting for the known prognostic variables age, FIGO stage, histological subtype and grade in the Cox model, VAV% had independent prognostic impact (HR 1.05, 95% CI 1.00 – 1.11, p=0.041), whereas age did not (p=0.40) (Table 4). There were no observed differences in the received surgical treatment (p=0.21) or adjuvant therapy (p=0.39) between patients with high versus low VAV% (data not shown).

**Table 4:** Prognostic impact of VAV% adjusted for FIGO stage, age, histological subtype and grade, for 225 endometrial cancer patients (Cox proportional hazards regression model)

	n	Unadjusted HR	95% CI	p-value	Adjusted HR	95% CI	p-value
**FIGO stage**				<0.001			**0.003**
Stage I	178	1			1		
Stage II	21	1.20	0.27 – 5.26	0.813	0.53	0.11 – 2.59	0.431
Stage III	21	6.61	2.85 – 15.29	<0.001	4.44	1.75 – 11.26	0.002
Stage IV	5	11.54	3.31 – 40.31	<0.001	3.11	0.80 – 12.10	0.101
**Histological subtype & grade**				<0.001			**0.001**
Endometrioid grade 1-2	152	1			1		
Endometrioid grade 3	32	2.22	0.68 - 7.20	0.185	1.12	0.31 - 4.06	0.860
Non-endometrioid	41	7.59	3.32 - 17.36	<0.001	4.91	1.94 - 12.42	0.001
**Age**	225	1.05	1.01 – 1.09	0.008	1.02	0.97 −1.07	0.400
**VAV%**	225	1.07	1.03 - 1.12	0.001	1.05	1.00 - 1.11	**0.041**

Abbreviations: CI: Confidence interval; FIGO: International federation of gynecology and obstetrics; HR: Hazard ratio; VAV%: Visceral fat percentage. p-value: Wald test.

### Global transcriptional analysis suggests increased immunogenic and inflammatory signaling in tumors arising in low VAV% context

Due to the observed survival differences, we further explored differences in gene expression patterns between tumors arising in a metabolic environment characterized by low versus high visceral fat percentage (VAV%). GSEA was performed, on the total series, and on endometrioid tumors separately. The tumors from patients with VAV% below median (<37%) showed enrichment of gene sets related to immune activation (e.g. immune response, T-cell activation, lymphocyte activation, response to bacterium) and inflammatory pathways (e.g. inflammatory response, interferon gamma response, cytokine binding), both when all histological types were included (Figure 1D, Supplementary Table 2), and in the subgroup of endometrioid tumors only (Supplementary Table 3). In tumors with high VAV%, false discovery rate (FDR) values tended to be higher, and thus no significantly enriched gene sets were identified in these tumors.

## DISCUSSION

Despite consistent evidence of increased endometrial cancer risk in obese women [1, 2], the underlying biological relation between obesity and the observed clinical and histopathological phenotypes in endometrial cancer is poorly understood. The present large endometrial cancer study links abdominal CT estimates reflecting obesity to a less aggressive histological phenotype, and suggests a negative prognostic impact of high visceral fat percentage (VAV%). Furthermore, low VAV% was linked to enrichment of gene sets regulating immunogenic and inflammatory response in the tumors. Thus, our data supports both a pathogenic and prognostic role of the metabolic environment induced by obesity in endometrial cancer, and suggests that transcriptional alterations in genes regulating immune- and inflammatory responses in the tumors may be linked to the metabolic environment in which the tumor arises.

This study shows that volumetric CT assessment of abdominal fat compartments and waist circumference provides quantitative obesity markers which are highly correlated with BMI and hepatic CT attenuation, which is a marker of steatosis (Table 2). To our knowledge, this is the first study of endometrial cancer presenting CT-based volumetric quantification of the abdominal fat compartments and estimates of liver density. Two previous CT studies on 122 [21] and 200 [22] EC patients, in which total, visceral and subcutaneous fat tissue areas were segmented on a single slice at the umbilical level, also showed positive correlations between BMI and total (r=0.87/0.67), subcutaneous (r=0.86/0.61) and visceral (r=0.75/0.43) fat areas (r=Pearson correlation coefficients for the two studies, respectively; all p-values <0.001). In our cohort, all the CT-derived obesity markers except VAV% were associated with low-grade endometrioid tumor subtype, which is clinically characterized by less aggressive disease. Our results are in line with the report by Nakamura *et al.*, except they found no association between histological subtype and visceral fat area [21].

Liver attenuation values have previously been shown to represent a fairly good radiologic estimate of hepatic steatosis [23]. We observed that decreasing hepatic attenuation was inversely correlated with BMI, TAV, SAV and VAV (Figure 1 and Table 2, all p<0.001), and also weakly negatively correlated with VAV% (p<0.05). The correlation coefficient was slightly stronger for VAV (ρ=-0.71) compared to SAV (ρ=-0.67); combined with the observed weak negative correlation between VAV% and LD (ρ=-0.16) our findings suggest that the visceral fat compartment may contribute slightly more than subcutaneous fat to obesity-associated hepatic steatosis. This is supported by a previous study reporting hepatic steatosis to be more closely linked to visceral adipose tissue than subcutaneous adipose tissue in healthy women [24].

To our knowledge, this is the first study presenting data on the relation between hormone receptor status in EC and CT-derived preoperative obesity markers. Interestingly, all the CT-estimated obesity markers except VAV% were associated with PR and AR positivity; both markers reportedly associated with less aggressive tumors [25, 26]. Furthermore, we observed a progressive loss of hormone receptor expression with decreasing total body fat (Figure 2), a trend that seems predominantly driven by PR loss. The present findings are in line with our previous report linking obesity to endometrioid histology and positive PR status [27]. Surprisingly, no significant association was observed between ERα status and obesity estimates, in spite of the fact that increased estrogen signaling is a well-established putative pathogenic mechanism in EC development in obese patients. This lack of association with ERα could be related to transcriptionally repressive effects on ERα mediated by PR signaling. In breast cancer cells, increased level of the PR B isoform has been reported to repress both ERα protein expression and mRNA levels [28] which is in line with our finding, but further studies are needed to understand the interplay between hormone receptor signaling in the setting of obesity in EC.

Previous reports on the impact of obesity, measured by BMI, on endometrial cancer survival are conflicting [27, 29–35]. Interestingly, whereas BMI, WC, LD and volumetric fat estimates were not associated with survival in this cohort, we found that patients with increasing visceral fat percentage (VAV%) had significantly reduced disease-specific survival, an effect that seemed to be independent of BMI (Table 4, Figure 1 and Supplementary Figure 2). Notably, disease-specific survival was intentionally chosen as end-point, thus ruling out other potential causes of early death reportedly linked to a high visceral fat proportion; i.e. cardiovascular disease, diabetes and other malignancies [11]. Based on the present finding, it seems reasonable to hypothesize that the metabolic environment induced by a high visceral fat proportion may be a driving factor for tumor progression and metastasis in endometrial cancer. Similar findings of reduced disease-specific survival in patients with high visceral fat proportion have been reported for other cancer types, including colorectal cancer [14, 15], esophageal cancer [13], lymphoma [36] and metastatic melanoma [12]. However, the relatively low number of events (n=28) in our cohort underlines that the independent prognostic impact of VAV% should be interpreted with care, and the finding needs to be validated in large independent endometrial cancer data sets in future studies. Nonetheless, CT-based estimates of abdominal fat volumes and fat distribution patterns represent promising biomarkers that may yield novel insight into the interplay between the metabolic environment and corresponding tumor biology. It should also be emphasized that these CT estimates are obtainable without much extra time-consume for the radiologist, and could thus quite easily be incorporated into the clinic.

Increasing VAV% was significantly associated with high age in our cohort. Similar findings were reported by Ye *et al.* [22], and the finding is in line with previous literature reporting gradual redistribution of fat to the visceral compartment with increasing age in healthy individuals [37]. Advanced age is known to be an unfavorable prognostic factor in EC [38]. Interestingly, when including both age and VAV% in the Cox model, only VAV% was an independent predictor of survival. Our results could thus suggest that the well-known unfavorable prognostic impact of advanced age in EC may in part be due to the metabolic effects mediated by the higher visceral fat proportion observed in elderly patients.

Although visceral adipose tissue is thought to promote carcinogenesis by inducing a state of low-grade systemic inflammation [11], we observed an opposite trend in tumor tissue: gene sets linked to inflammation and immune response were enriched in tumors arising in patients with low VAV%, who had better survival compared to patients with high VAV%. In line with this, high epithelial infiltration of CD8+ T-lymphocytes has been reported to be associated with favorable EC prognosis [39, 40]. Our finding of improved outcome in patients with low VAV%, may thus perhaps be explained by a more preserved tumor immune response. Also, in a recent study of tumor-bearing mice, systematic physical exercise reportedly led to reduced tumor size with upregulation of genes associated with immune activity [41]. This study also supports the notion that the metabolic environment in which a tumor arises may influence host defense responses, which may ultimately have an impact on tumor growth and patient survival. However, functional studies are needed to reveal the relevant mechanisms involved, and are necessary if novel targets for treatment are to be identified.

This study has some limitations. Our estimates of hepatic attenuation were performed on contrast-enhanced CT scans which were carried out as a part of the routine diagnostic work-up. Although contrast-enhanced CT scans have been shown to provide good measures of hepatic steatosis, non-contrast-enhanced CT is considered slightly better and is regarded as the gold standard for evaluation of hepatic steatosis [23]. We were not able to adjust for alcohol consumption or other potential confounding factors related to increased hepatic steatosis since this information was not available. Also, our BMI calculations (measured at time of diagnosis) and all CT-based estimates only provide a snapshot of the metabolic status at one particular time-point of the disease. We did not have any data regarding history of weight gain/weight loss, or changes in fat distribution over time prior to disease development; factors that potentially also could be linked to tumor development and survival. To be able to better adjust for potential confounding factors, a larger study including information for these factors is needed.

In conclusion, this study suggests a negative prognostic impact of high visceral fat percentage (VAV%) in EC. Tumors arising in patients with low VAV% showed enrichment of gene sets related to increased immunogenic and inflammatory signaling, thus supporting that the metabolic environment in which the tumor arises may influence its immune response. Altered tumor immune response in relation to obesity should be further explored, and may reveal possible therapeutic targets for novel treatment of endometrial cancer patients.

## MATERIALS AND METHODS

### Patient series

We included 227 women diagnosed with primary endometrial cancer between 2009 and 2014 at Haukeland University Hospital, Bergen, Norway. The patient series was extracted from a larger, population based, prospectively collected and well annotated endometrial cancer cohort, the Momatec (Molecular Markers in Treatment of Endometrial Cancer) study, previously well described [25, 42]. All participants signed informed consent, and the local ethical committee approved the study (REK numbers 2015/2333 and 2009/2315). All the included patients had undergone either preoperative abdominal contrast-enhanced computed tomography (CT) scans (n=225) or non-contrast enhanced CT scans (n=2), as part of the routine diagnostic work-up. Primary treatment consisted of hysterectomy with bilateral salpingo-oophorectomy (HBSO) in 97% of the cases (221/227). If HBSO was not performed (6/227), the patients were staged based on results from curettage, clinical examination and preoperative imaging. All patients were staged according to the FIGO 2009 criteria [43]. Clinicopathological and follow-up data were collected by review of the medical records. The median follow-up time of survivors was 4.1 years (range 0.1 – 7.6), and patients were followed from the date of primary surgery until December 22^nd^ 2016, or death. Histological type was classified as endometrioid or non-endometrioid, the latter including serous-papillary tumors (n=22), clear cell tumors (n=5), carcinosarcomas (n=10) and undifferentiated tumors (n=4). BMI was calculated from measured height and weight at the time of diagnosis (weight in kg divided by height in meters squared). Patient characteristics are summarized in Table 1. For a subset of the included patients, immunohistochemical data for estrogen receptor alpha (ERα; n=170), progesterone receptor (PR; n=170) and androgen receptor (AR; n=169) expression were available, with complete overlap between the three hormone receptors for 149 patients. The staining procedure and evaluation for ERα [44, 45], PR [26] and AR [25] has been described previously. Ploidy status, estimated by flow cytometry as previously described [46], was available for 119 patients. For 105 patients, fresh frozen tissue was available for global gene expression analyses, further described below.

### Image analyses

Diagnostic abdominal contrast-enhanced CT scans (n=225) and non-contrast CT scans (n=2) were evaluated for assessment of abdominal fat volume. By the software iNtuition (TeraRecon Inc., San Mateo, CA, USA), cross-sectional images were analyzed consecutively from the upper right diaphragm to L5/S1-level, using a semi-automated method for volumetric quantification of abdominal fat [17]. This method is based on segmentation of pixels with values for Hounsfield units (HU) corresponding to adipose tissue (−195 to −45 HU) [17]. The correct segmentation of the subcutaneous and visceral fat compartments was visually verified by the operator, and manually adjusted if necessary (Figure 1A and 1B). The visceral abdominal fat volume (VAV; cm^3^) and subcutaneous abdominal fat volume (SAV; cm^3^) were estimated. The two compartments were considered to comprise the total abdominal fat volume (TAV, cm^3^). The percentage of visceral out of total abdominal fat volume ([VAV/TAV]x100; VAV%) was also calculated. Waist circumference (WC; cm) was measured at the level of vertebral body L3/L4.

To estimate liver steatosis as a surrogate marker of obesity [3, 20], we used the software ImageJ [47] to register attenuation values measured in Hounsfield units (HU) on contrast-enhanced images in portal venous contrast phase [23]. Mean hepatic parenchymal attenuation values were measured in three distinct circular regions of interest (ROI) with a diameter of 15 millimeters, and the mean value of the three ROIs was calculated and used in the analyses. Care was taken to avoid inclusion of visually distinct vasculature or biliary ducts, and focal liver lesions, if present, in the ROIs. Two patients did not have available contrast-enhanced CT scans, and liver density measurement was not performed.

### Expression microarrays and data analysis

RNA was extracted from fresh frozen tumor tissue, and hybridized to Agilent Whole Human Genome Microarray Kit, 44k (catalogue number G4 112F) as described previously [44]. Arrays were scanned using the Agilent Microarray Scanner Bundle. The software J-express (www.molmine.com) [48] was used for data analysis. Median spot intensity was used to define the intensity signal, and the data set was checked for batch effects before and after quantile normalization. Gene set enrichment analysis (GSEA) (www.broadinstitute.org/gsea) [49] was performed on the expression data set collapsed to max probe (total 30,500 probes), using the Molecular Signatures Database (MSigDB, version 5.1) datasets Hallmark and c5 (Gene ontology gene sets) (www.broadinstitute.org/gsea/msigdb). A false discovery rate (FDR) < 5% was set as cut-off level when determining significantly differentially expressed gene sets between patients with high versus low VAV%.

### Statistical analysis

Statistical analyses were performed using SPSS (Statistical Package of the Social Sciences), version 23.0 (IBM Corp, 2015). Correlations were assessed by Spearman's rank correlation (ρ=rho). To compare the distribution of a continuous variable between two groups, the Mann-Whitney U-test was applied, and between multiple groups the Kruskal-Wallis H test or Jonckheere-Terpstra trend test was used. For analyses of disease specific survival (DSS), patients were included at the date of primary surgery, and the primary endpoint was defined as death from endometrial cancer. Patients who died from other causes were censored at the date of death. To examine if any of the obesity variables were associated with survival, the Cox Proportional Hazards Regression Model was used, after visual assessment of included variables by a log-minus-log plot to check the assumption of proportional hazards. Only univariable significant predictors were included in the final multivariable Cox-model. VAV% was dichotomized according to median for visual presentation of survival data in a Kaplan-Meier plot, assessing survival differences between groups by the two-sided log-rank test (Mantel-Cox). This dichotomization was also used when comparing differentially expressed gene sets between groups (high versus low VAV%). All statistical tests were two-sided, and p-values less than 0.05 were considered significant.

## SUPPLEMENTARY MATERIALS FIGURES AND TABLES







## Abbreviations

AR: Androgen receptor
BMI: Body mass index
CT: Computed tomography
DSS: Disease specific survival
EC: Endometrial cancer
ERα: Estrogen receptor alpha
FDR: False discovery rate
FIGO: International Federation of Gynecology and Obstetrics
GSEA: Gene set enrichment analysis
HBSO: Hysterectomy with bilateral salpingo-oophorectomy
HR: Hazard ratio
HU: Hounsfield units
LD: Liver density
PR: Progesterone receptor
ROI: Region of interest
SAV: Subcutaneous abdominal fat volume
TAV: Total abdominal fat volume
VAV: Visceral abdominal fat volume
VAV%: Visceral fat percentage
WC: Waist circumference
