# Supplementary material for: High visceral fat percentage is associated with poor outcome in endometrial cancer
Source: Oncotarget. 2017 Oct 19;8(62):105184–95. doi: 10.18632/oncotarget.21917 (PMC5739630; doi:10.18632/oncotarget.21917)
Supplement: Supplementary file 1 [file oncotarget-08-105184-s001.pdf]

High visceral fat percentage is associated with poor outcome in endometrial cancer

SUPPLEMENTARY MATERIALS

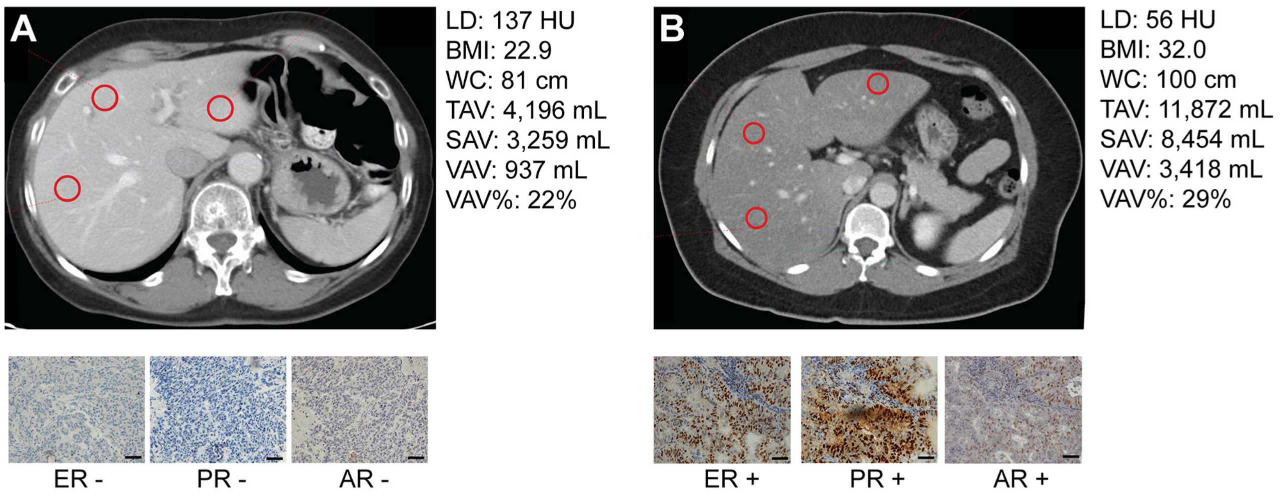

Supplementary Figure 1: Hepatic attenuation measurement on contrast-enhanced CT scans and immunohistochemical staining of corresponding tumor tissue. Three distinct regions of interest (ROIs) (red circles) were drawn on representative parts of the liver parenchyma, and mean density in Hounsfield units (HU) measured. Abdominal CT at the level of the liver in two different patients with high and low liver density are shown in panels A and B, respectively. Immunohistochemical (IHC) staining of the corresponding tumour tissue for estrogen receptor (ER), progesterone receptor (PR) and androgen receptor (AR) are shown below. Panel **(A)** negative ER, PR and AR staining. Panel **(B)** positive ER, PR and AR staining. Bar scale for IHC stainings: 100 µm.

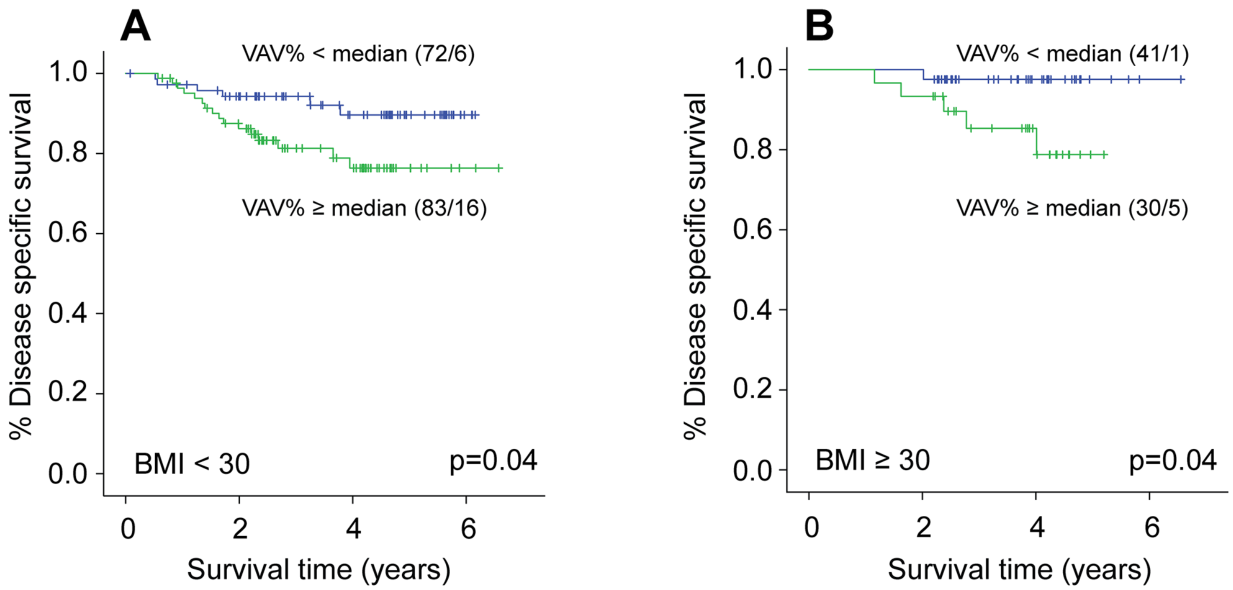

Supplementary Figure 2: Prognostic effect of VAV% in BMI subgroups. Kaplan-Meier curve showing the prognostic effect of visceral fat percentage (VAV%) in subgroups of patients according to BMI. **(A)** Survival according to VAV% median (37%) for 155 patients with BMI < 30 (p=0.04). **(B)** Survival according to VAV% median (37%) for 71 patients with BMI ≥ 30 (p=0.04). P-values: log-rank test.

Supplementary Table 1: BMI and CT-estimated obesity parameters in relation to clinicopathological factors and hormone receptor status for 186 endometrioid endometrial cancer patients

See Supplementary File 1

Supplementary Table 2: Top ranked gene sets with false discovery rate (FDR) <5% from GSEA comparing patients with high (≥37%) versus low (<37%) VAV% in 105 endometrial tumors. Category indicates whether gene set is (1) linked to processes related to inflammation and immune activation or (2) other cellular processes

Rank	Gene sets enriched in tumors with low VAV% (n=56)	FDR (%)	Category
	Hallmark gene sets		
1	HALLMARK_ALLOGRAFT_REJECTION	0	1
2	HALLMARK_IL6_JAK_STAT3_SIGNALING	0	1
3	HALLMARK_INFLAMMATORY_RESPONSE	0	1
4	HALLMARK_ESTROGEN_RESPONSE_EARLY	0.04	2
5	HALLMARK_REACTIVE_OXIGEN_SPECIES_PATHWAY	0.13	2
6	HALLMARK_ESTROGEN_RESPONSE_LATE	0.11	2
7	HALLMARK_MTORC1_SIGNALING	0.16	2
8	HALLMARK_PI3K_AKT_MTOR_SIGNALING	0.80	2
9	HALLMARK_CHOLESTEROL_HOMEOSTASIS	0.96	2
10	HALLMARK_INTERFERON_GAMMA_RESPONSE	0.87	1
11	HALLMARK_ANDROGEN_RESPONSE	0.82	2
12	HALLMARK_APOPTOSIS	1.20	2
13	HALLMARK_FATTY_ACID_METABOLISM	1.57	2
14	HALLMARK_BILE_ACID_METABOLISM	1.46	2
15	HALLMARK_XENOBIOTIC_METABOLISM	1.70	2
16	HALLMARK_PEROXISOME	1.88	2
17	HALLMARK_GLYCOLYSIS	2.28	2
18	HALLMARK_IL2_STAT5_SIGNALING	2.29	1
19	HALLMARK_UNFOLDED_PROTEIN_RESPONSE	3.32	2
20	HALLMARK_TNFA_SIGNALING_VIA_NFKB	3.54	1
21	HALLMARK_HYPOXIA	4.34	2
22	HALLMARK_HEME_METABOLISM	4.64	2
	Gene ontology (GO) gene sets		
1	DEFENSE_RESPONSE	1.53	1
2	IMMUNE_RESPONSE	1.06	1
3	CELLULAR_DEFENSE_RESPONSE	1.02	2
4	IMMUNE_SYSTEM_PROCESS	2.63	1
5	T_CELL_ACTIVATION	2.36	1

(Continued)

Rank	Gene sets enriched in tumors with low VAV% (n=56)	FDR (%)	Category
6	INTERLEUKIN_RECEPTOR_ACTIVITY	2.64	1
7	ECTODERM_DEVELOPMENT	2.90	2
8	OXIDOREDUCTASE_ACTIVITY_GO_0016705	2.55	2
9	EPIDERMIS_DEVELOPMENT	2.48	2
10	G_PROTEIN_COUPLED_RECEPTOR_BINDING	2.51	2
11	INTERLEUKIN_BINDING	2.65	1
12	LYMPHOCYTE_ACTIVATION	2.59	1
13	INFLAMMATORY_RESPONSE	2.48	1
14	RESPONSE_TO_BACTERIUM	2.34	1
15	LEUKOCYTE_ACTIVATION	2.32	1
16	RESPONSE_TO_BIOTIC_STIMULUS	2.46	1
17	CHEMOKINE_RECEPTOR_BINDING	2.38	1
18	CYTOKINE_BINDING	2.53	1
19	INTEGRAL_TO_ORGANELLE_MEMBRANE	3.13	2
20	CELL_ACTIVATION	3.91	2
21	CHEMOKINE_ACTIVITY	4.13	1
22	CORNIFIED_ENVELOPE	4.26	2
23	DEFENSE_RESPONSE_TO_BACTERIUM	4.88	1

Supplementary Table 3: Top ranked gene sets with false discovery rate (FDR) <5% from GSEA comparing patients with high (≥37%) versus low (<37%) VAV% in 85 endometrioid tumors. Category indicates whether gene set is (1) linked to processes related to inflammation and immune activation or (2) other cellular processes

See Supplementary File 2
