# Supplementary material for: High visceral fat percentage is associated with poor outcome in endometrial cancer
Source: Oncotarget. 2017 Oct 19;8(62):105184–95. doi: 10.18632/oncotarget.21917 (PMC5739630; doi:10.18632/oncotarget.21917)
Supplement: Supplementary file 2 [file oncotarget-08-105184-s002.docx]

| **Supplementary Table 1:** BMI and CT-estimated obesity parameters in relation to clinicopathological factors and hormone receptor status for 186 endometrioid endometrial cancer patients | | | | | | | | | | | | | | | | |
| --- | --- | --- | --- | --- | --- | --- | --- | --- | --- | --- | --- | --- | --- | --- | --- | --- |
|  |  |  | **BMI** | | **WC** | | **TAV** | | **VAV** | | **SAV** | | **VAV%** | | **LD** | |
|  |  | **n (%)** | **median** | **p** | **median** | **p** | **median** | **p** | **median** | **p** | **median** | **p** | **median** | **p** | **median** | **p** |
|  |  |  | **(kg/m^2^)** |  | **(cm)** |  | **(ml)** |  | **(ml)** |  | **(ml)** |  | **(%)** |  | **(HU)** |  |
| **Histological grade** *(n=184)* | |  |  | **0.04** |  | NS |  | **0.02** |  | **0.02** |  | **0.01** |  | NS |  | **0.04** |
|  | Grade 1-2 | 152 (83) | 28.0 |  | 98 |  | 10,302 |  | 3,647 |  | 6,032 |  | 37 |  | 99 |  |
|  | Grade 3 | 32 (17) | 25.2 |  | 87 |  | 6,645 |  | 2,241 |  | 4,310 |  | 36 |  | 109 |  |
| **FIGO stage** | |  |  | NS |  | NS |  | NS |  | NS |  | NS |  | NS |  | NS |
|  | I+II | 169 (91) | 26.8 |  | 96 |  | 9,548 |  | 3,480 |  | 5,934 |  | 37 |  | 101 |  |
|  | III+IV | 17 (9) | 26.8 |  | 96 |  | 9,732 |  | 3,295 |  | 5,042 |  | 40 |  | 91 |  |
| **Ploidy** *(n=86)* | |  |  | NS |  | NS |  | NS |  | NS |  | NS |  | NS |  | NS |
|  | Diploid | 75 (87) | 27.8 |  | 96 |  | 10,046 |  | 3,533 |  | 5,809 |  | 37 |  | 98 |  |
|  | Aneuploid | 11 (13) | 26.7 |  | 99 |  | 9,864 |  | 4,363 |  | 5,389 |  | 44 |  | 91 |  |
| **Age (median)** | |  |  | **0.03** |  | NS |  | NS |  | NS |  | **0.02** |  | **<0.001** |  | NS |
|  | < 67 years | 95 (51) | 28.0 |  | 96 |  | 9,459 |  | 3,354 |  | 6,120 |  | 34 |  | 98 |  |
|  | ≥ 67 years | 91 (49) | 26.7 |  | 96 |  | 9,732 |  | 3,725 |  | 5,453 |  | 40 |  | 101 |  |
| **ERα expression, IHC** *(n=137)* | |  |  | NS |  | NS |  | NS |  | NS |  | NS |  | NS |  | NS |
|  | Positive | 105 (77) | 27.1 |  | 98 |  | 10,290 |  | 3,552 |  | 5,809 |  | 37 |  | 100 |  |
|  | Negative | 32 (23) | 26.4 |  | 96 |  | 9,134 |  | 3,100 |  | 5,924 |  | 36 |  | 101 |  |
| **PR expression, IHC** *(n=138)* | |  |  | **<0.001** |  | **<0.001** |  | **<0.001** |  | **<0.001** |  | **<0.001** |  | NS |  | **0.01** |
|  | Positive | 118 (86) | 28.2 |  | 99 |  | 10,507 |  | 3,771 |  | 6,232 |  | 37 |  | 98 |  |
|  | Negative | 20 (14) | 23.9 |  | 86 |  | 5,713 |  | 2,030 |  | 3,313 |  | 38 |  | 111 |  |
| **AR expression, IHC** *(n=136)* | |  |  | **0.02** |  | **0.01** |  | **0.01** |  | **0.01** |  | **0.02** |  | NS |  | **0.04** |
|  | Positive | 91 (67) | 29.1 |  | 100 |  | 10,660 |  | 3,970 |  | 6,284 |  | 37 |  | 96 |  |
|  | Negative | 45 (33) | 25.4 |  | 91 |  | 7,554 |  | 2,877 |  | 4,730 |  | 37 |  | 105 |  |
| Abbreviations: AR: Androgen receptor; BMI: Body mass index; CT: Computed tomography; ERα: Estrogen receptor; FIGO: International federation of gynecology and obstetrics; HU: Hounsfield units; IHC: Immunohistochemistry; LD: Liver density; NS: Not significant; PR: Progesterone receptor; p: p-values (Independent samples Kruskal-Wallis test for Histologic subtype and grade, remaining p-values: Mann-Whitney U test); SAV: Subcutaneous abdominal fat volume; TAV: Total abdominal fat volume; VAV: Visceral abdominal fat volume; VAV%: Visceral fat percentage; WC: Waist circumference. | | | | | | | | | | | | | | | | |
| Liver density: data missing for one patient. | | | | | | | | | | | | | | | | |
