# Supplementary material for: High visceral fat percentage is associated with poor outcome in endometrial cancer
Source: Oncotarget. 2017 Oct 19;8(62):105184–95. doi: 10.18632/oncotarget.21917 (PMC5739630; doi:10.18632/oncotarget.21917)
Supplement: Supplementary file 3 [file oncotarget-08-105184-s003.docx]

| **Supplementary Table 3:** Top ranked gene sets with false discovery rate (FDR) <5% from GSEA comparing patients with high (≥37%) versus low (<37%) VAV% in 85 endometrioid tumors. Category indicates whether gene set is (1) linked to processes related to inflammation and immune activation or (2) other cellular processes | | | |
| --- | --- | --- | --- |
| **Rank** | **Gene sets enriched in tumors with low VAV% (n=40)** | **FDR (%)** | **Category** |
|  | ***Hallmark gene sets*** |  |  |
| 1 | HALLMARK_ALLOGRAFT_REJECTION | 0 | 1 |
| 2 | HALLMARK_IL6_JAK_STAT3_SIGNALING | 0 | 1 |
| 3 | HALLMARK_INFLAMMATORY_RESPONSE | 0 | 1 |
| 4 | HALLMARK_INTERFERON_GAMMA_RESPONSE | 0 | 1 |
| 5 | HALLMARK_MTORC1_SIGNALING | 0.04 | 2 |
| 6 | HALLMARK_COMPLEMENT | 0.04 | 1 |
| 7 | HALLMARK_REACTIVE_OXIGEN_SPECIES_PATHWAY | 0.03 | 2 |
| 8 | HALLMARK_FATTY_ACID_METABOLISM | 0.03 | 2 |
| 9 | HALLMARK_PI3K_AKT_MTOR_SIGNALING | 0.05 | 2 |
| 10 | HALLMARK_IL2_STAT5_SIGNALING | 0.15 | 1 |
| 11 | HALLMARK_APOPTOSIS | 0.14 | 2 |
| 12 | HALLMARK_TNFA_SIGNALING_VIA_NFKB | 0.15 | 1 |
| 13 | HALLMARK_XENOBIOTIC_METABOLISM | 0.25 | 2 |
| 14 | HALLMARK_UNFOLDED_PROTEIN_RESPONSE | 0.33 | 2 |
| 15 | HALLMARK_PEROXISOME | 0.48 | 2 |
| 16 | HALLMARK_INTERFERON_ALPHA_RESPONSE | 0.49 | 1 |
| 17 | HALLMARK_ADIPOGENESIS | 0.76 | 2 |
| 18 | HALLMARK_HEME_METABOLISM | 1.19 | 2 |
| 19 | HALLMARK_ANDROGEN_RESPONSE | 1.16 | 2 |
| 20 | HALLMARK_UV_RESPONSE_UP | 1.53 | 2 |
| 21 | HALLMARK_KRAS_SIGNALING_UP | 1.47 | 2 |
| 22 | HALLMARK_CHOLESTEROL_HOMEOSTASIS | 1.92 | 2 |
| 23 | HALLMARK_EPITHELIAL_MESENCHYMAL_TRANSITION | 2.11 | 2 |
| 24 | HALLMARK_BILE_ACID_METABOLISM | 2.33 | 2 |
| 25 | HALLMARK_ESTROGEN_RESPONSE_EARLY | 2.56 | 2 |
| 26 | HALLMARK_OXIDATIVE_PHOSPHORYLATION | 3.27 | 2 |
| 27 | HALLMARK_ANGIOGENESIS | 4.46 | 2 |
|  | ***Gene ontology (GO) gene sets*** |  |  |
| 1 | DEFENSE_RESPONSE | 0 | 1 |
| 2 | IMMUNE_RESPONSE | 0 | 1 |
| 3 | LYMPHOCYTE_ACTIVATION | 0 | 1 |
| 4 | IMMUNE_SYSTEM_PROCESS | 0 | 1 |
| 5 | CELLULAR_DEFENSE_RESPONSE | 0.06 | 2 |
| 6 | LEUKOCYTE_ACTIVATION | 0.05 | 1 |
| 7 | T_CELL_ACTIVATION | 0.04 | 1 |
| 8 | INFLAMMATORY_RESPONSE | 0.04 | 1 |
| 9 | CELL_ACTIVATION | 0.03 | 1 |
| 10 | CYTOKINE_BINDING | 0.36 | 1 |
| 11 | RESPONSE_TO_EXTERNAL_STIMULUS | 0.33 | 1 |
| 12 | POSITIVE_REGULATION_OF_TRANSLATION | 0.3 | 2 |
| 13 | INTERLEUKIN_BINDING | 0.29 | 1 |
| 14 | CELLULAR_RESPIRATION | 0.32 | 2 |
| 15 | INTERLEUKIN_RECEPTOR_ACTIVITY | 0.34 | 1 |
| 16 | G_PROTEIN_COUPLED_RECEPTOR_BINDING | 0.45 | 2 |
| 17 | RESPONSE_TO_BIOTIC_STIMULUS | 0.43 | 2 |
| 18 | COFACTOR_METABOLIC_PROCESS | 0.51 | 2 |
| 19 | SUGAR_BINDING | 0.48 | 2 |
| 20 | ELECTRON_CARRIER_ACTIVITY | 0.49 | 2 |
| 21 | LOCOMOTORY_BEHAVIOR | 0.46 | 2 |
| 22 | MULTI_ORGANISM_PROCESS | 0.51 | 2 |
| 23 | AEROBIC_RESPIRATION | 0.48 | 2 |
| 24 | CYTOKINE_PRODUCTION | 0.67 | 1 |
| 25 | CHEMOKINE_RECEPTOR_BINDING | 0.87 | 1 |
| 26 | POSITIVE_REGULATION_OF_CYTOKINE_BIOSYNTHETIC_PROCESS | 0.88 | 1 |
| 27 | REGULATION_OF_LYMPHOCYTE_ACTIVATION | 0.92 | 1 |
| 28 | CYTOKINE_BIOSYNTHETIC_PROCESS | 0.95 | 1 |
| 29 | CYTOKINE_METABOLIC_PROCESS | 1.01 | 1 |
| 30 | ALDO_KETO_REDUCTASE_ACTIVITY | 1.13 | 2 |
| 31 | ADAPTIVE_IMMUNE_RESPONSE_GO_0002460 | 1.22 | 1 |
| 32 | SULFUR_COMPOUND_BIOSYNTHETIC_PROCESS | 1.22 | 2 |
| 33 | REGULATION_OF_CYTOKINE_BIOSYNTHETIC_PROCESS | 1.19 | 1 |
| 34 | RESPONSE_TO_WOUNDING | 1.41 | 1 |
| 35 | ADAPTIVE_IMMUNE_RESPONSE | 1.57 | 1 |
| 36 | COENZYME_METABOLIC_PROCESS | 1.62 | 2 |
| 37 | ANTIGEN_BINDING | 1.62 | 1 |
| 38 | CHEMICAL_HOMEOSTASIS | 1.62 | 2 |
| 39 | POSITIVE_REGULATION_OF_MULTICELLULAR_ORGANISMAL_PROCESS | 1.61 | 2 |
| 40 | CATION_HOMEOSTASIS | 1.65 | 2 |
| 41 | CHEMOKINE_ACTIVITY | 1.76 | 1 |
| 42 | OXIDOREDUCTASE_ACTIVITY | 1.75 | 2 |
| 43 | DETECTION_OF_BIOTIC_STIMULUS | 1.97 | 2 |
| 44 | DETECTION_OF_STIMULUS | 1.95 | 2 |
| 45 | OXIDOREDUCTASE_ACTIVITY_ACTING_ON_THE_CH_CH_GROUP_OF_DONORS | 1.93 | 2 |
| 46 | MITOCHONDRION_ORGANIZATION_AND_BIOGENESIS | 1.93 | 2 |
| 47 | PHAGOCYTOSIS | 1.96 | 2 |
| 48 | HUMORAL_IMMUNE_RESPONSE | 1.94 | 1 |
| 49 | OXIDOREDUCTASE_ACTIVITY_GO_0016705 | 1.90 | 2 |
| 50 | ION_HOMEOSTASIS | 1.87 | 2 |
| 51 | HEME_METABOLIC_PROCESS | 1.84 | 2 |
| 52 | CELLULAR_CATION_HOMEOSTASIS | 1.86 | 2 |
| 53 | STEROID_BIOSYNTHETIC_PROCESS | 1.86 | 2 |
| 54 | T_CELL_DIFFERENTIATION | 1.89 | 1 |
| 55 | PATTERN_RECOGNITION_RECEPTOR_ACTIVITY | 2.20 | 1 |
| 56 | METALLOENDOPEPTIDASE_ACTIVITY | 2.25 | 2 |
| 57 | POSITIVE_REGULATION_OF_LYMPHOCYTE_ACTIVATION | 2.41 | 1 |
| 58 | LIPID_METABOLIC_PROCESS | 2.52 | 2 |
| 59 | CYTOKINE_SECRETION | 2.51 | 1 |
| 60 | DETECTION_OF_EXTERNAL_STIMULUS | 2.48 | 2 |
| 61 | MEMBRANE_ORGANIZATION_AND_BIOGENESIS | 2.72 | 2 |
| 62 | RESPONSE_TO_OTHER_ORGANISM | 2.77 | 1 |
| 63 | RESPONSE_TO_BACTERIUM | 2.79 | 1 |
| 64 | APOPTOSIS_GO | 2.85 | 2 |
| 65 | PROGRAMMED_CELL_DEATH | 2.87 | 2 |
| 66 | POSITIVE_REGULATION_OF_CELLULAR_PROTEIN_METABOLIC_PROCESS | 3.07 | 2 |
| 67 | POSITIVE_REGULATION_OF_IMMUNE_SYSTEM_PROCESS | 3.05 | 1 |
| 68 | BEHAVIOR | 3.16 | 2 |
| 69 | INTEGRAL_TO_ORGANELLE_MEMBRANE | 3.12 | 2 |
| 70 | CELLULAR_HOMEOSTASIS | 3.15 | 2 |
| 71 | OXIDOREDUCTASE_ACTIVITY_GO_0016616 | 3.37 | 2 |
| 72 | ELECTRON_TRANSPORT_GO_0006118 | 3.73 | 2 |
| 73 | VIRAL_REPRODUCTION | 3.68 | 1 |
| 74 | REGULATION_OF_IMMUNE_SYSTEM_PROCESS | 3.63 | 1 |
| 75 | MITOCHONDRIAL_TRANSPORT | 3.59 | 2 |
| 76 | HOMEOSTATIC_PROCESS | 3.60 | 2 |
| 77 | RAS_PROTEIN_SIGNAL_TRANSDUCTION | 3.76 | 2 |
| 78 | EXTRACELLULAR_SPACE | 3.78 | 2 |
| 79 | POSITIVE_REGULATION_OF_PROTEIN_METABOLIC_PROCESS | 3.99 | 2 |
| 80 | MEIOTIC_CELL_CYCLE | 3.99 | 2 |
| 81 | CELLULAR_LIPID_METABOLIC_PROCESS | 3.94 | 2 |
| 82 | STEROID_METABOLIC_PROCESS | 3.93 | 2 |
| 83 | PIGMENT_METABOLIC_PROCESS | 3.91 | 2 |
| 84 | CARBOHYDRATE_BINDING | 4.05 | 2 |
| 85 | IMMUNE_EFFECTOR_PROCESS | 4.17 | 1 |
| 86 | ENDOPLASMIC_RETICULUM_MEMBRANE | 4.12 | 2 |
| 87 | ENDOPLASMIC_RETICULUM_PART | 4.20 | 2 |
| 88 | SOLUBLE_FRACTION | 4.36 | 2 |
| 89 | REGULATION_OF_T_CELL_ACTIVATION | 4.34 | 1 |
| 90 | OXIDOREDUCTASE_ACTIVITY_ACTING_ON_CH_OH_GROUP_OF_DONORS | 4.35 | 2 |
| 91 | REGULATION_OF_APOPTOSIS | 4.55 | 2 |
| 92 | REGULATION_OF_PROGRAMMED_CELL_DEATH | 4.86 | 2 |
| 93 | INTRINSIC_TO_ORGANELLE_MEMBRANE | 4.83 | 2 |
| 94 | NITROGEN_COMPOUND_METABOLIC_PROCESS | 4.79 | 2 |
| 95 | REGULATION_OF_I_KAPPAB_KINASE_NF_KAPPAB_CASCADE | 4.94 | 1 |
| 96 | IMMUNOLOGICAL_SYNAPSE | 4.90 | 1 |
| 97 | POSITIVE_REGULATION_OF_T_CELL_ACTIVATION | 4.99 | 1 |
| 98 | COFACTOR_BIOSYNTHETIC_PROCESS | 4.99 | 2 |
